# Introducing the Research Design of Phenomenography

**DOI:** 10.1007/s40670-024-02082-0

**Published:** 2024-07-23

**Authors:** Danica Anne Sims

**Affiliations:** 1 Faculty of Education, University of Oxford, Oxford, UK; 2 Biomedical Engineering and Healthcare Technology (BEAHT) Research Centre, Faculty of Health Sciences, University of Johannesburg, Johannesburg, South Africa

**Keywords:** Conceptions, Methodology, Phenomenography, Qualitative research, Research design

## Abstract

This article introduces the lesser known qualitative research design of phenomenography to medical science and health professions education researchers. Phenomenography, as distinct from phenomenology, seeks to describe and organise the different ways people experience and understand a phenomenon. Here, the origins and philosophical underpinnings of phenomenography are briefly shared, and an outline of how a phenomenographic research study may be undertaken is presented. This includes data collection tools, analytic method, and examples from the field. Overall, phenomenography is valuable for better understanding the varied experiences of students, educators, practitioners, and patients, with implications for pedagogy, practice, and related outcomes.

## Introduction

This paper introduces readers to the research approach of phenomenography [1–3], which is not to be confused with the better known methodology of phenomenology. This introduction outlines what phenomenography is and how to do it, as a relevant, but underutilised research design in medical sciences and health professions education [4, 5]. Phenomenography explores the diversity of understandings of social phenomena in natural settings, as phenomena are inherently perceived in varied and multidimensional ways; in other words, people experience the same phenomenon in different, and partial or incomplete, ways [6, 7]. Phenomenography describes and organises variations in experiences and subsequent understandings, of students, educators, researchers, patients, and health professionals, which can have implications for educational, clinical, and healthcare system practice, management, development, and change [4, 5]. For instance, in educational contexts, understanding students' diverse experiences and conceptualisations of learning and different subject matters, can inform how educators design curricula to address different levels of understanding, and to more effectively teach students, through correcting misconceptions or tailoring communication and instructional strategies. 

## What Is Phenomenography?

**Fig. 1:**
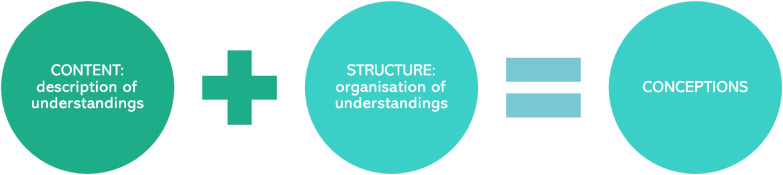
A conception is both the varied description of an experience and subsequent understanding (the content or ‘what’ of conceptions), as well as its organisation or relationship towards other descriptions (the structure or ‘how’ of conceptions)

Phenomenography is a qualitative research approach^1^ that is situated within an interpretative or constructivist research paradigm^2^ [4, 8]. According to its Greek origins, phenomenography means ‘description of appearances’ [3]. These ‘description of appearances’ are called *conceptions* [2, 3, 9]. Conceptions must be distinguished from other terms that are often used in the literature, including experiences, perspectives, perceptions, ways of seeing, views, thoughts, beliefs, orientations, mental models or structures, mindsets, personal theories or philosophies, and metaphors. While these terms may overlap with conceptions in terms of describing different ways of experiencing and understanding, they fail to capture the *structural* aspect of phenomenography—the organisation of these different ways of experiencing and understanding (see analysis below and Fig. 1).

**Fig. 2:**
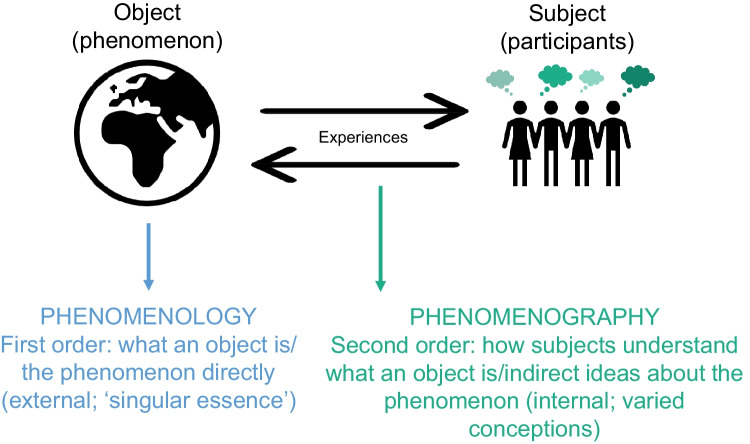
Phenomenology versus phenomenography: first- and second-order perspectives. Phenomenography describes an aspect of the world as it appears to a group of individuals at a particular time, how they explain it to themselves and others [2, 3, 5], whereas phenomenology is concerned with the phenomenon itself [2, 10]

Conceptions have been clearly defined as the *qualitatively different ways* individuals’ *experience* and *understand*, or make meaning of, a phenomenon [2, 3]. Conceptions are a collection of different, but related, ‘cognitive representations’ of reality and ‘intellectual maps’ of experiences [5]. If ‘concepts’ may be described as basic units of thought, then conceptions can be thought of as the varied designs of thoughts (understandings or qualifications of the concept), conceptualised or created by the human mind based on their experiences of said concept [10]. This is why conceptions can be described as ‘relational knowledge’, for they are rooted in the ‘experience-concept duo’ [10] (see Fig. 2).

Phenomenography should therefore be used when wanting to describe *variation* in lived experiences and resultant *understandings* of a single (the same) phenomenon (not multiple or interrelated phenomena) by a *group* of people [6]. Table 1 offers possible types of appropriate research questions phenomenographic researchers can ask [10], and Table 2 provides real-world examples of phenomenographic research questions from the field.

**Table 1:** Examples of types of phenomenographic research questions [10]

• What are [subjects’] experiences of [concept/phenomenon] and in what ways and degrees do they differ?
• What is the diversity and depth of experience-based conceptualisations of [subjects] about [concept/phenomenon]?
• How and in which hierarchical steps, and to what extent, do the conceptualisation of [subjects’] regarding [concept/phenomenon] diversify?
• What are the dimensions and depths of [subjects’] understandings regarding [concept/phenomenon]?
• How and what ways do [subjects] experience and understand [concept/phenomenon]?

**Table 2:** Examples of phenomenographic research in health professions education: questions and results

• How do medical students conceptualise learning for written examinations: tactical memorising, comprehensive, application-directed, and holistic approaches [11]
• How medical students understand anatomy: through contextualisation, visualisation, selection, and anatomical language [12]
• How do clinical supervisors understand what it means to be a good teacher: conveying knowledge, responding to student content requests, showing how things are done, modelling what is means to be a doctor, and developing student growth [13]
• How do nurse educators conceptualise their teaching and learning roles: initiating, supporting, becoming part of, and owning these roles [14]
• How do dental and medical clinician educators understand their development as educators: educator and clinician roles as separate, the educator role as embedded in the clinician role, and confidence in both roles [15]
• How do occupational therapy supervisors conceptualise trust: trust is static and about the student, trust is dynamic and based on student performance, and trust as mutual and interrelated [16]
• How do nursing students, supervisors, and clinician educators conceptualise the purposes of clinical learning: to meet curricular demands, to deliver patient care, to deliver patient care within the larger (healthcare) context, and to continuously develop as a professional [17]
• How do doctoral nursing students conceptualise their learning: as preparation for nursing, as problematising practical problems against scientific theories, and as a transformative process of developing from nurse to researcher [18]
• How do nursing students conceptualise competence: as task completion, as passing assessments and satisfying facilitators, as applying theory to practice, as performance of nursing according to clinical standards, and as performance that yields positive health outcomes [19]

Originally, in the 1970s, in Sweden, in the field of Higher Education, Marton [1–3] and colleagues developed phenomenography to understand the varied ways university students approach their learning (as surface or deep approaches) and the related differences in their learning outcomes, which had implications for curriculum development, and teaching and learning strategies. This ‘shallowness’ and ‘depth’ of understanding, to be expanded upon later, becomes an important feature of phenomenography [1–3, 10]. Table 2 provides examples of more recent phenomenographic research from the field of medical and health professions education. Note the range and diversity of understandings, and how some may appear somewhat simplistic and one-dimensional, and others more sophisticated and complex, indicating both differences in experiences, as well as the relationships or progression between different conceptions (Table 2).

Phenomenography is often confused with the more popular and well-used methodology of phenomenology. While both seek to interpret and understand lived experiences, phenomenology is interested in the world as it *is* and phenomenography the world as *perceived* [5]. Put differently, phenomenology explores *first-*order perspectives and phenomenography *second*-order perspectives (Fig. 2). First-order perspectives describe the *object* or phenomenon *directly* as it seeks to develop a common or constant description of *what* something *is* at its essential core (its central structure or the ‘inherent essence’ of a phenomenon) - transcending its subjective experience [20]. Second-order perspectives describe how the *subject* *interprets* the object or phenomenon, i.e. *how* people diversely experience, interpret, and *understand* what something is - embracing subjective plurality [2, 21]. Additional differences between phenomenography and phenomenology are their emphases: phenomenography is interested in *collective* meanings and phenomenology *individual* experiences (termed ‘lifeworld’) [5–8]. Moreover, phenomenography is concerned with *hierarchically diagramming* different ways of experiencing and their related understandings, the shallowness or depth of those understandings (i.e. the relationships between conceptions and their organisation or progression), where phenomenology is not [2, 6]. To illustrate, if assessment in medical sciences was the phenomenon of interest, phenomenology would ask, ‘What is assessment?’, whereas phenomenography would ask, ‘What are the different ways people experience and understand what assessment is?’

Ontologically, phenomenography is situated within a *non-dualist ontology*, meaning that there is no separation between internal/subjective and external/objective - it is not purely objective (independent of people) nor purely subjective (independent of the world) [21, 22]. In short, the only world we can communicate is the world as we experience it [23]. This connects with aforementioned descriptions of conceptions as ‘relational knowledge’ and the ‘experience-concept duo’ [10]: meaning is constructed through the *relationship* (i.e. experience) between the subject/individual and the object/phenomenon [21]. Epistemologically, as variation of understandings is the focus of phenomenography, and indeed we differ in our experiences and thus knowledges of the world, multiple subjective knowledges are seen valid [23].

## How to Undertake a Phenomenographic Research Study

### Sampling

**Fig. 3:**
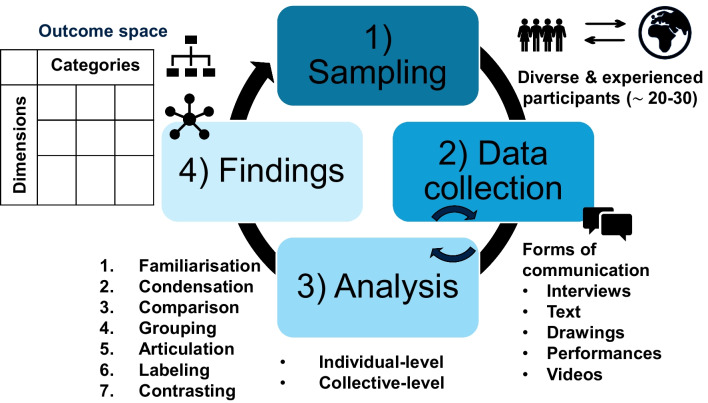
An overview of a phenomenographic research study: sampling, data collection, analysis, and findings

What phenomena can be researched using phenomenography? Anything that may lead to a conceptual differences or variations in understandings [10]. Therefore, to capture these differences, sampling should include participants that are both experienced in the phenomenon of interest and purposefully diverse or heterogeneous, as opposed to inexperienced and homogenous populations (Fig. 3) [10].

When it comes to determining the number of participants needed, a helpful starting point can be referring to sample size ranges in published phenomenographic studies (e.g. from the studies in Table 2, the number of participants ranged from 8 to 48), with a general recommendation being 20 to 30 participants [24]. However, and as mentioned in the introduction, individuals experience phenomena in partial or incomplete ways; consequently, this means that until iterative data collection and analysis are underway, and the depth, quality, and completeness or holism of the data becomes clearer, it can be difficult to determine how many participants are sufficient beforehand. Related to rigour below, information power, conceptual depth, and theoretical sufficiency are useful criteria to consider during data collection and analysis to evaluate if sampling is adequate and data collection can cease [25].

### Data Collection

The goal of data collection in phenomenography is to *jointly* explore the experience under investigation as *deeply* and *fully* as possible. Data is most often collected through interviews, along with lesser used written text or other forms of communication (e.g. drawings, performances, video, focus groups, observations, questionnaires, think-aloud methods) [5]. As with any rigorous qualitative research study, the data collection tools selected must be fit for purpose, aligning with the research question, to provide relevant and useful data. General principles for in-depth qualitative research interviews apply to phenomenographic interviews: developing and piloting the interview guide, considering dimensions of culture and power, building rapport, and co-constructing data with participants, attentive listening, and timely transcription and analysis [26, 27].

More specifically, phenomenographic interviews should be semi-structured, with questions related to the phenomenon under investigation, but still flexible and open-ended to avoid limiting the dialogue [3, 4, 28]. Participants should be able to dynamically and democratically dictate the interviews, with subsequent questioning, clarifying, and prompting following up on their responses, instead of rigidly following a predetermined list of questions and answers [4, 7, 21, 28]. Immediately asking abstract questions such as ‘What do you understand about [the concept/phenomenon]’ can be unproductive and inaccessible, as participants can become confused or not understand the question. Instead, participants can be asked to describe their concrete actions related to the phenomenon, and then reflect on their experiences, followed by more specific and abstract prompting (e.g. How…? Why…? What did you mean…? Can you explain…?), to enable more accessible and meaningful data collection, and allow the researcher works backwards to uncover underlying conceptions [29].

The general principles and practices of quality, rigour, and trustworthiness (i.e. credibility, transferability, dependability, confirmability) for qualitative research studies [8, 30, 31] apply to phenomenographic research too. For instance, reflexivity^3^ [32] which relates to ‘bracketing’ for unbiased data collection and interpretation [7, 28, 33, 34]. Bracketing is when the researcher suspends their own presuppositions and experiences of the phenomenon, and what other research findings, evidence, literature, authorities, or theories may say, so that the *participant’s* understandings are genuinely captured [28, 33]. There are, however, debates around bracketing, if it is necessary or even possible [28], as well as the relationship and interpretative awareness between the researcher, the phenomenon, and data [6], yet this is beyond the scope of this piece.

### Analysis

Data is analysed through a coding process similar to Braun and Clarke’s popular thematic analysis [35]: familiarisation of data (verbatim transcription, repeated reading and reviewing of the dataset), identification and condensation of units of meaning or central ideas (coding), comparison of similarities and differences of codes, grouping of codes into categories (common or shared expressions of understandings), articulating the essential meaning or description of each category, labelling categories, and contrasting categories to determine how they differ and are distinct from each other [4, 23, 36]. Participant quotations are used to illustrate and support findings [23], aligning with second-order perspectives (i.e. the subject’s own accounts of their experiences) [28]. This further supports the authenticity and rigour of proposed findings [8, 23].

Analysis is also performed at two levels: meaning within the *individual* transcripts and the ‘pools of meanings’ across the *collective* dataset (all transcripts together). This has been described as a ‘zigzag’ process [10], which represents the iterative nature of qualitative research. By analysing at both levels, researchers can ensure that their interpretations remain true to the participant, but also extend to the group and broader context of meaning, as individuals rarely express complete conceptions, but highlight different dimensions or critical variations that are important to them [5]. More importantly, phenomenography does not describe individual’s different experiences, but the *collective* variation in experiences of a phenomenon *holistically* [22]. Logically, if there are a finite number of ways something can be experienced, sharing experiences across individuals is then expected. Similarly, a single individual cannot exhaustively explain all aspects of a phenomenon but experience it partially. Therefore, the *sum* or *whole* of the understandings at a *group level* is necessary and a distinguishing feature of phenomenography [27].

In contrast to other qualitative methods of analysis (e.g. thematic or content analysis), a unique feature of phenomenography is that conceptions are not simply broad themes but *organised* descriptions of meaning [4]. To use phenomenographic speak, conceptions encompass both the *meaning*, or content, of understandings (i.e. a *what* attribute), and the *structure*, or relationships, between the different ways of understanding (i.e. a *how* attribute) (see Fig. 1) [9, 28]. To demonstrate this structure and the relationships between different understandings (how they are ‘internally related’), conceptions are depicted in a typology (classification system) called an *outcome space* [4, 5]. An outcome space is a diagrammatic representation of the logically structured constructions of understanding at a collective level, depicting the full range of possible ways of experiencing the phenomenon under investigation [2, 3, 37]. Outcome spaces classify previously unspecified ways of thinking about a phenomenon by explicitly describing the *differing contents* and *levels of understandings*. The ‘levels’ of understandings relates to previous comments around the shallowness versus depth of understandings. Outcome spaces can take different forms, for example, a two-dimensional table or branching mind map.

### Representing Findings

Outcome spaces consist of *categories* and *dimensions* and represent the ‘whole’ of the phenomenon as experienced and thus conceptualised. Categories are distinctly grouped ‘descriptions of understanding’, representing the central, and distinctive, meaning of a conception at a collective level [4, 7]. Categories are described and distinguished from each other by the dimensions, with relationships between different categories clearly logical [4, 7]. Dimensions are the salient features, the specific descriptors, or characteristics, assigned to each conception and variation [37] (see Table 3).

**Table 3:**
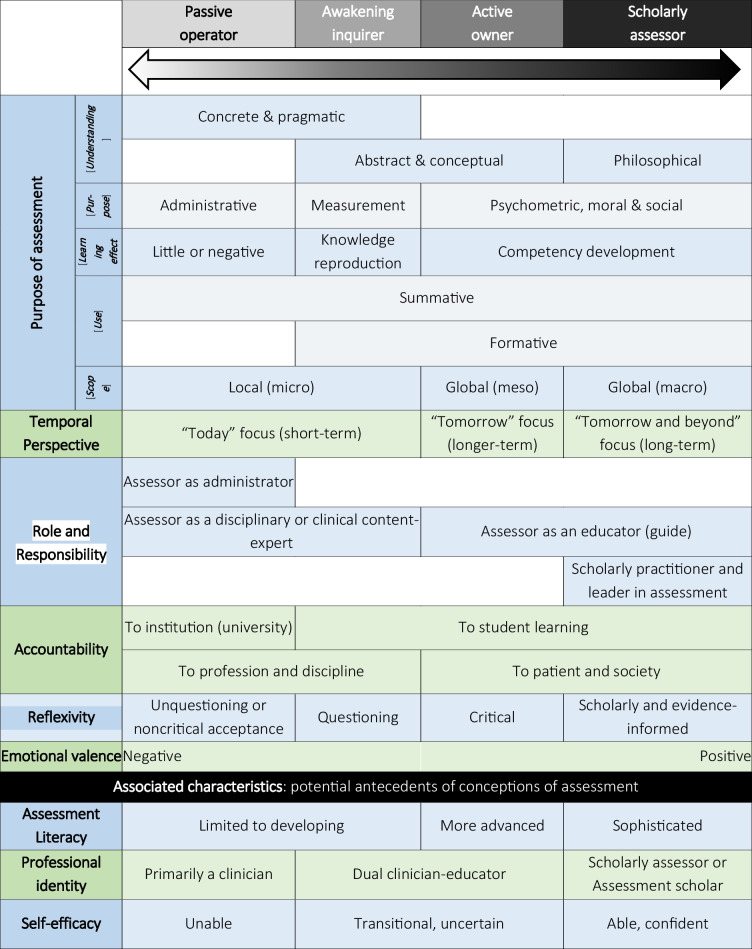
An example of an outcome space depicting clinician educators’ conceptions of assessment, hierarchically arranged, in undergraduate medical programmes (adapted from [38])

The outcome space reveals that there are a limited number of ways (categories) a phenomenon can be qualitatively experienced [27, 28]. The classification (organisation) of these descriptions into categories reveals the structurally significant differences between ways of understanding. While each category is distinct, the outcome space is *logically related* and *hierarchical*—there is a progression of understanding through the outcome space [28]. Simply put, conceptions generally exist along a spectrum or a continuum from more basic, simplistic, superficial and incomplete understandings at one end to more complex, comprehensive, sophisticated, and advanced understandings at the other end (i.e. from ‘low’ to ‘high’ levels of understanding) [27]. This means that subsequent categories encompass and build upon previous categories (e.g. like an inverted pyramid or expanding concentric circles). For this reason, an outcome space has been described as ‘hierarchically inclusive’ [4, 38].

A critique of phenomenography and its outcome space is the idea that some conceptions, on the ‘lower’ end of the spectrum, can be seen as deficient or deviant versions of an ‘ideal’ conception [28]. Value judgements of ‘worse’ or ‘better’ understandings is not the purpose of phenomenography, rather *describing variation* is the goal [22]. Instead, outcome spaces should be seen in developmental, not deficit, terms, indicating differential conceptual starting points that are open to potential growth and progression. Conceptions are not static, but dynamic, and able to evolve over time and space [21]. This makes sense as conceptions are rooted in experience: additional experiences (e.g. educational interventions) are likely to influence or change said conceptions.

For example, I have used phenomenography to explore the different ways clinician educators understand assessment in undergraduate medical programmes (Table 3) [38]. The idea was that we first needed to know how these individuals experienced and understood assessment, and how these understandings may have influenced their assessment practices, before interventions like targeted faculty development could be implemented to more effectively enhance their assessment thinking and related assessment practices. By offering personalised educational experiences, meeting individuals at their different conceptual starting points, we would then hope to shift their understandings, moving their conceptions up and along the outcome space, leading to an enhanced assessment thinking and practice.

## Conclusion

Lastly, while there is greater complexity and debate around phenomenography, and variations in practice, this introductory piece does not seek to paint a complete or an exhaustive picture; rather, I hope that the utility and value of this research design has been outlined for interested researchers to investigate further. In closing, phenomenography holds immense promise for medical sciences and health professions education research for the improved understanding of varied educational and clinical experiences of students, educators, patients, and health professionals in evolving and diverse educational and healthcare contexts. Practically, as links between conceptions and practice have been observed, implications for developing understanding and related pedagogical and professional practices, and their outcomes, are supported.

## Footnotes

1 Qualitative research seeks to explore and interpret complex phenomena and human experience through in-depth analysis of non-numerical data (e.g., interviews, observations, artefacts) and emphasises the context and meaning from the subjective perspective of the participants.

2 A paradigm is a lens or framework through which the world is viewed and research conducted. Paradigms consist of sets of beliefs, and assumptions, about the world: ontology (nature of reality), epistemology (nature of knowledge), and methodology (nature of research). An interpretative or constructivist paradigm views reality as subjectively experienced, socially constructed and manifold, knowledge as subjective and individual, with methodologies selected to interact with others to construct knowledge and better understanding these multiple subjective experiences. See: Brown, M.E.L. and A.N. Duenas, *A Medical Science Educator’s Guide to Selecting a Research Paradigm: Building a Basis for Better Research.* Med Sci Educ, 2019. **30**(1): p. 545–553.

3 Reflexivity in research is an active and ongoing critical self-awareness and mitigation of potential harmful subjectivities (e.g., preconceptions, prejudices, biases, stereotypes, and beliefs held by the researcher), accounting for the possible influence of and distortion by the researcher on the research process and its outcomes. Reflexivity relates to the process of ‘bracketing’.
